# NEWS

**DOI:** 10.7189/jogh.05.010201

**Published:** 2015-06

## AFRICA

➢ Almost 4000 Liberians died in the recent Ebola outbreak, but thanks to behavioural changes and massive international aid efforts, there are only six confirmed cases of Ebola left in the country. As a result, commercial activity is returning to normal, and schools and hospitals are beginning to re–open. Stephen Kennedy, a Liberian doctor, symbolises the new spirit of hope and resilience in Liberia. He is spear–heading a new vaccine trial that could prevent future outbreaks. “I feel so excited. This is something I have dreamed of – to help my country make a major contribution to international public health,” he says. (*BBC*, 2 Feb 2015)

➢ Sierra Leone’s national auditor, Lara Taylor–Pearce, states that government ministers lost US$ 3 million of internal emergency funds to fight Ebola, with another US$ 2.5 million incompletely accounted for. This led to “a reduction in the quality of service delivery in the health sector.” These funds were from taxes and donations from individuals – mainly in Sierra Leone – and excludes UN and aid agency funding. According to the BMJ, one–third of the US$ 2.9 billion pledged by international donors had not reached Sierra Leone, Guinea and Liberia by the end of 2014; and the UN is seeking an additional US$ 1 billion of aid to contain Ebola. (*New York Times*, 14 Feb 2015)

➢ The South African research consortium Follow–on Africa Consortium for Tenofovir Studies (FACTS) found that a microbicide gel believed to reduce the risk of a woman being infected with HIV during sex is ineffective. This in contrast to a 2010 study which suggested the gel reduced infection rates by 39%. The e gel contains the antiretroviral compound tenofovir, and is applied to the rectum or vagina to protect against HIV and other sexually transmitted infections. There are currently no commercially–available microbiocide gels, although WHO figures show that 23 are being clinically tested. The gel’s effectiveness could be undermined by application problems – emphasizing the need for women–centred HIV–prevention methods. (*Mail and Guardian*, 23 Feb 2015)

➢ Hifikepunye Pohamba, Namibia’s outgoing President, won the Mo Ibrahim Prize for “good governance” in Africa. The US$ 5 million is given to a democratically–elected former head of state who left office in the previous 3 years, and has demonstrated “exceptional leadership” whilst abiding by constitutional term limits. Mr. Pohamba has been lauded for reconciling with opponents, pushing for gender equality in politics, and increased expenditure on housing and education. Salim Ahmed Salim, chair of the prize committee, singled out Mr. Pohamba’s “sound and wise leadership” and “humility” during his presidency. The prize had only been awarded on three occasions, due to many African leaders seeking to stay in power as long as possible. (*yahoo news*, 3 March 2015)

➢ The African Union (AU) criticised the international media for overlooking Africa’s efforts to tackle Ebola, instead focusing on international agencies and those with the greatest media clout. Dr Olawale Maiyegun, director of Social Affairs at the AU, claimed that Africa had taken the lead in the most critical human resources for health (it deployed 835 health workers to the affected countries), whilst most of the international community focused on finance and infrastructure efforts. However, the AU has faced criticism for holding an emergency summit 10 months after the outbreak of Ebola. Dr Maigegun responded by saying that the AU’s response was guided by the affected countries. “We put volunteers at the disposal of the affected countries. They told us what to do and we have performed creditably,” he said. (*The Guardian*, 7 April 2015)

## ASIA

➢ In Nov 2014, Indonesia’s new government (led by President Joko Widodo) began issuing cards to give poor Indonesians access to expanded publicly–funded health care and education programmes, and access to an income top–up scheme worth US$ 15.75 a month. The top–up scheme will eventually reach one–third of Indonesia’s population – or 86.4 million people – making it the largest such programme in the world, and could lift millions out of poverty. The programme is financed by ending petrol subsidies, which disproportionately benefitted wealthier Indonesians. Although there are logistical challenges in providing services across such a vast dispersed country, and in using mobile technology to collect top–up payments, in the long–term the scheme could make Indonesians healthier and better–educated – and ultimately wealthier. (*The Economist*, 8 Jan 2015)

➢ The Population Council Pakistan carried out two studies on the country’s induced abortions and unintended pregnancies, and low rates of contraceptive use in collaboration with the Guttmacher Institute USA. They uncovered rising rates of induced abortions and unintended pregnancies in women aged 15–49 years, jumping from 27 per 1000 women in 2002 to 50 per 1000 in 2012. In 2012 alone, 4.2 million out of 9 million pregnancies were unintended, with 54% ending in abortion and 623 000 women being treated for post–abortion complications. This is against a background of low rates of contraceptive usage, and high discontinuation rates. The studies’ partners say that the unmet need for birth control was a major cause of increasing abortion rates – risking women’s lives – and there is a real need to reduce the gap between the supply and demand for contraceptive services. (*The Express Tribune*, 29 Jan 2015)

➢ According to the World Economic Forum (WEF), Indonesia is now South East Asia’s largest economy, but non–communicable diseases (NCDs) threaten it with a US$ 1 trillion burden – more than five times its 2012 GDP. In 2014, deaths from NCDs accounted for 71% of all deaths – up from 51% in 2004. The WEF believes that the ageing Indonesian population and rapid urbanisation – with its associated prevalence of risk factors like tobacco, harmful alcohol use, poor diet and sedentary lifestyles – underpins this increase. Indonesia’s output loss from NCDs is higher than China and India. However, investing in health interventions to reduce NCDs have high returns, both socially and economically. (*CNBC*, 20 Apr 2015)

➢ Cambodia is one of the world’s fastest–growing economies, with average growth at 7% per annual, income levels of nearly US$ 1000 per capita, and poverty has fallen from 50% of the population to 20%. Economic growth has been accompanied by major improvements in the country’s health – during 2000 – 2012, life expectancy increased from 61.9 years to 71.4 years, the infant mortality rate fell from 95 to 28, the under–5 mortality rate declined from 124 to 35 during 2000 – 2012, maternal mortality rates fell from 437 to 170, and attended births rose from 50% to 89%. Many communicable diseases (eg, HIV, malaria) are largely under control. However, major challenges remain, with disparities between urban and rural areas, and health outcomes do not yet match other countries in the region. Poor water and sanitation, high neonatal mortality and malnutrition persist. The government is providing health equity funds to enable free access to health services for those in need, and 2 million people are now covered. The country’s health planners aims to extend health coverage, improved training and licensing of health workers, and stronger monitoring and evaluation. (*SciDev.net*, 23 Apr 2015)

➢ The death toll from the earthquake which struck Nepal has passed 7 500, and will become higher as rescue teams reach isolated villages. Aid agencies report that Nepalese local governments are overwhelmed, as it has been 80 years since the country’s last comparable disaster. Médecins Sans Frontičres (MSF) report that more aid is reaching areas outside the capital, Kathmandu, as the challenges from getting aid from the bottlenecks at Kathmandu ease. According to the UN, 8 million out of Nepal’s 28 million people were affected by the earthquake, and at least 2 million need tents, water, food and shelter. “Our priority is to reach people in places where no one else is going and who have not received assistance. So it has been a huge challenge logistically to get the necessary supplies in through the congested airport, and secure the air transport we need to be able to provide medical assistance and deliver shelter and relief materials to the people in most urgent need,” says MSF’s Dr Prince Mathew. (*Irish Times*, 6 May 2015)

## AUSTRALIA AND WESTERN PACIFIC

➢ Refugees from Africa, the Middle East and South Asia who are held on the Pacific island of Nauru after being declined by Australia could be offered payment by the Australian government to settle in Cambodia. This is part of a wider Australian policy of deterring refugees from reaching Australia by boat, and is condemned by human right activities as inhumane and potentially dangerous. As yet, no refugee has accepted the offer, which includes cash, help in finding work, access to education, language training, accommodation and health insurance. (*ABC News*, 17 Apr 2015)

➢ Australia removed a religious exemption that allowed parents unwilling to immunise their children from claiming certain benefits, and unveiled a US$ 20 million package to increase child vaccination rates. More than 39 000 children under 7 years were not immunised due to the parents’ objections – an increase of 24 000 over the past 10 years. Australia has a vaccination rate of over 90% for children aged 1–5 years, and in 2014 at least 166 000 children were recorded as overdue for immunisation for more than two months. Other measures to boost immunisation include the establishment of a national school vaccination register and financial incentives for doctors to follow–up on children late for vaccinations. An anti–vaccination movement has coincided with increasing cases of measles in parts of Europe and the USA. Many people avoid immunisation for their children over concerns over the triple vaccines for measles, mumps and rubella alleged link to autism – a theory which has been repeatedly disproven. (*AFP*, 19 Apr 2015)

➢ The New Zealand government is facing accusations of reneging on an election promise to provide free GP visits for primary school children. Documents obtained by the country’s Green Party show that the government is funding only 90% of doctors’ visits for children suffering from an injury. Government ministers replied by stating that they expect uptake to eventually become universal, and that it has provided an additional US$ 21 million of funding to doctors to encourage them to provide free visits for children aged under 13 years. Moreover, some doctors in wealthier areas charge patients an additional fee on top of their government payment. (*stuff.co.nz*, 21 Apr 2015)

➢ Healthy volunteers are taking part in a trial at the University of Queensland, where they are infected with low doses of malaria to test the efficacy of new drug treatments. This accelerates the development of new drugs, saving money in R&D costs and provides hope for the development of more effective vaccines. Under this testing model, volunteers are injected with a sample of malaria that is much smaller than that transmitted by a mosquito bite. They are closely monitored using tests to measure the DNA of malaria parasites. This allows researchers to treat them before they become ill. Researchers can administer trial drugs to monitor their effectiveness, and capture information that can help develop treatments. This enables researchers to know quickly if a drug is suitable for large–scale trials, and provides data to support vaccine development. “It is important that we understand the human biology of the vaccines before we start doing big clinical trials,” says Tim Wells, Chief Scientific Officer at Medicines for Malaria Venture. (*Financial Times*, 23 Apr 2015)

➢ UNICEF Australia has criticised new cuts to Australia’s international aid programmes, which total US$ 800 million. UNICEF and other aid agencies will have to scale–back operations as a result. Australia’s contribution to international aid already falls short of the UN’s target of 0.7% of GDP. Felicity Weaver, UNICEF Australia’s International Programme Manager, says that these cuts are unfair. She cited how the cholera outbreak in Zimbabwe cost 4000 lives, and that US$ 800 000 cuts to a programme to install and manage clean water for small communities in Zimbabwe is risking lives. (*UNICEF Australia*, 13 May 2015)

## CHINA

➢ A study by New York University and East China Normal University (*Walking, obesity and urban design in China)* uncovers the impact of the built environment on physical activity in six densely–populated areas in Shanghai and Hangzhou. The areas were rated for ease of walking and cycling, including footpaths, trees, benches, street width and kerb cuts; plus barriers to exercise such as pavement and bicycle lane obstructions and air pollution. It found that rates of walking and cycling for recreation and commuting are higher in areas with fewer impediments. Although the results may be unsurprising, they could be crucial in persuading local government officials and developers of the benefits of more walkable urban development patterns – at a time of high and increasing rates of obesity and chronic diseases in Chinese cities. (*Cities Today*, 3 Mar 2015)

➢ China has set out a roadmap for fixing its health care system, which is plagued by queues and poor rural services. China’s State Council announced plans to double the number of general doctors to two per 1000 people by 2020, and increase the numbers of nurses and support staff. It will also investigate using technology such as mobile devices and online cloud systems to support these changes, and introduce databases for electronic health records and expand patient information records across the entire population. It will speed up the development of grassroots health care, and may further open up health care to the private sector. China’s health care market will be worth an estimated US$ 1 trillion by 2020, making is highly lucrative for drug manufacturers, medical device firms and hospital operators. (*Reuters*, 31 Mar 2015)

➢ Lao People’s Democratic Republic began a GAVI–supported campaign to vaccinate its children about Japanese Encephalitis (JE). This campaign marks the first time GAVI has provided support to a Chinese–manufactured vaccine – made possible by WHO’s decision to pre–qualify the vaccine produced by the Chengdu Institute of Biological Product. This is the first time a Chinese manufacturer has achieved this standard. JE, a vaccine–preventable disease, is mosquito–borne with no specific treatment – and can lead to severe disability or death. Each year, there are 70 000 cases of JE, and 20 000 resulting deaths. WHO estimates that 3 billion people in Asia are at risk of JE, and 24 countries have endemic transmission. (*Financial Express*, 1 Apr 2015)

➢ Alibaba Groups Holdings is expanding into China’s booming online health care markets. It announced a US$ 2.5 billion deal to move its web pharmacy business into its publicly listed health division. This will enable the group to move into the sale of prescription drugs if regulations change, and places it to cater to an aging, wealthier population wrestling with chronic conditions (eg, heart disease, diabetes), that is more interested in health products and monitoring. Expanding China’s online drug sales could reshape its US$ 149 billion pharmaceuticals market by moving sales online away from hospitals, and its fragmented pharmaceutical supply chain offer further opportunities for efficiency gains. (*bloomberg.com*, 15 Apr 2015)

➢ The Hong Kong government has proposed new regulations on the nutrition and health claims on infant formula milk, aiming to increase breast–feeding rates. To date, Hong Kong has been lax in allowing manufacturers to make misleading claims on the benefits of formula–feeding, which may cause some mothers to believe that formula milk is better than breast milk. The WHO recommends breastfeeding for at least the first six months of a child’s life. The government has yet to decide whether to ban all nutrient and health claims, or whether to allow some health claims. Manufacturers’ associations are campaigning for more flexibility, whilst breastfeeding campaigners argue for a more restrictive approach. Any restriction would apply to infant formula, follow–up formula and pre–packaged food for children aged under 36 months. (*South China Morning Post*, 17 Apr 2015)

## EUROPE

➢ Cancer Research UK forecasts that 1–in–2 people in the UK will be diagnosed with cancer at some point in their lives – an increase from its earlier estimate of 1–in–3 people. Increased life–expectancies, and fewer deaths from infections and heart disease mean that more people are living long enough to develop cancer. However, cancer survival rates are increasing, and improving lifestyle (eg, losing weight and stopping smoking) have major benefits. Breast and prostate cancers are likely to remain the most common, although head and neck cancers (caused by the human papillomavirus) and tumours in the food pipe (linked to acid reflex causes by obesity) are becoming more common. Sean Duffy, the national clinical director for cancer at NHS England, calls for improved prevention, faster diagnosis, better treatment, and care and after–care for all patients. (*BBC News*, 4 Feb 2015)

➢ A study of 26 European countries, published in the *BJOG: An International Journal of Obstetrics & Gynaecology*, found wide variations in Caesarean section (C–section) rates across Europe. Rates are 17% in Sweden, 25% in the UK and 52% in Cyprus. It found that C–section rates have risen in most EU countries, although may be levelling off in some countries. C–sections are safe procedures, albeit riskier than vaginal births. Reasons behind this increase may include a fear of litigation, financial incentives, maternal requests, and the belief that it is a safe procedure. Prof. Alison Macfarlane, professor of perinatal health at City University London said “given that people are supposed to be practicing according to evidence, it is surprising there is such wide variations between countries,” and called for further research to ensure that clinical practice is based on evidence. (*BBC*, 9 Mar 2015)

➢ Overall, cases of TB in Europe and Central Asia fell by 5.6% between 2012 and 2013, although prevalence is still high in certain countries, and globally the region is the most affected by multi–resistance TB. In 2013, the region had 360 000 reported cases of TB, with 85% occurring in 18 “high priority” countries in Eastern Europe and Central Asia (eg, Romania, Turkey, Kazakhstan, Russia etc.). The incidence of TB is higher where national incomes are lower, and/or where income inequalities are higher. Europe will not be free of TB this century at the current rate of eradication, and the WHO calls for governments to scale up access to effective treatments, and improve diagnosis and care. TB is the second most deadly infectious disease after AIDS, with 1.5 million deaths in 2013 compared to 1.6 million AIDS–related deaths in 2012. (*AFP*, 17 Mar 2015)

➢ In an open letter to the WHO Regional Office for Europe, the European Public Health Alliance (EPHA) states its strong support for the WHO’s nutrient profile model. It recognises the model as an invaluable tool in identifying foods high in fat, salt and sugar (HFSS), which will curb the marketing of unhealthy foodstuffs to children and ultimately decrease child obesity. EPHA states that voluntary action is inadequate to control the marketing of HFSS food and beverages to children due to wide variations in practice and compliance, and calls for formal regulation. (*EPHA*, 14 Apr 2015)

➢ The UN’s Sustainable Development Solutions Network shows that Switzerland tops its World Happiness index, closely followed by Iceland, Denmark, Norway and Canada. The World Happiness Report, which aims to influence government policy, bases its rankings on variables such as real GDP per capita, healthy life expectancy, corruption levels and social freedoms. It found the least happy countries were Togo, Burundi, Syria, Benin and Rwanda. (*BBC News*, 24 Apr 2015)

## INDIA

➢ India’s human development lags behinds its economic development, illustrated by having one of the highest rate of infant deaths in the world – three times higher than China. It spends just over 1% of GDP on publicly–funded health care, compared to 2.9% in China and 4.1% in Brazil, and every year 60 million Indian people are pushed into poverty due to health care costs. The government is designing a National Health Assurance Mission to provide universal health care. To succeed, it must increase expenditure from US$ 20 billion to US$ 45 billion, focusing on high impact investments that could bring India’s rate of preventable infectious, maternal and child deaths in line with other middle–income countries, eg, China, Cuba etc. India must expand the use of current medicines, vaccines and diagnostic tests, invest in maternal, newborn and child health, immunisations, malaria control, health–systems strengthening and tackling TB. Action such as tobacco taxation is also needed to tackle non–communicable diseases. The economic payoff would be vast – an estimated US$ 10 for every US$ 1 invested. (*The Economic Times India*, 12 Dec 2014)

➢ HIV support groups claim that India’s state–run HIV/AIDS programme (National AIDS Control Organisation – NACO) has ran out of critical supplies, leaving tens of thousands of people without access to life–saving drugs. Supplies of testing kits are also at low levels, and there are reports of difficulties in drug users obtaining clean syringes, and high–risk groups obtaining free condoms. NACO has succeeded in reducing new HIV infections from 274 000 in 2000 to 116 000 in 2011, although India has the 3rd highest number of cases in the world with 2.1 million people living with HIV. “People are being left to die on the road because of bureaucratic delays,” says Ms Kousalya Periaswamy, founder of the Positive Women Network and one of the first Indian women to openly state her HIV–positive status. (*WSJ*, 12 Feb 2015)

➢ The Institute of Palliative Medicine has extended its community Palliative Care project across Kerala, Tamil Nadu, Puducherry and West Bengal, caring for terminally–ill people who were not receiving care from their communities or medical system. The project uses village–based volunteers who provide care – spanning medical, emotional, spiritual, financial, familial and logistical care – to dying people, as well as fund–raising to support the project’s work. Approximately 100 000 volunteers provide sensitive, compassionate and competent care to people with terminal or chronic illnesses. Sivanthi, a volunteer, brings her children on home visits, and says it has instilled a strong feeling of compassion in her children. (*The Epoch Times*, 15 Feb 2015)

➢ India’s child health – still poor despite the country’s robust economic growth – is a major public health issue. A child raised in India is much more likely to be malnourished than a child from DR Congo, Zimbabwe or Somalia, with poor sanitation and increasing drug–resistant infections affecting nutrition. Another important factor is the relatively poor health of Indian women. More than 90% of adolescent girls are anaemic (a key measure of poor nutrition), and 42% of Indian mothers are underweight, compared to 16.5% in sub–Saharan Africa. Indian children are also smaller than their counterparts in sub–Saharan Africa. This is surprising, as on average, Indian people are wealthier, better educated and have fewer children. However, gender differences in employment, education and status (eg, Indian women will often eat less than other family members) are wider in India, and this plays a part in malnourished mothers and small children. (*New York Times*, 2 Mar 2015)

¬ Each year, 59 000 people die from rabies transmitted by dogs, with developing countries the worst affected. Rabies is a fatal infection which is almost 100% preventable. Most developed countries have eliminated rabies from their dog populations, but rabies is present in domestic dogs in developing countries, and is often poorly controlled. Most deaths occur in Asia and Africa, with India alone accounting for 35% of deaths. This represents an economic cost of US$ 8.6 billion through premature deaths and lost income. The Global Alliance for Rabies Control calls for increased efforts to vaccinate dogs, particularly in low–income countries, and for vaccines for bite victims to be more affordable and more available. (*BBC News*, 17 Apr 2015)

## THE AMERICAS

➢ The US pharmaceutical company Pfizer announced that it will cut the price of its pneumococcal vaccine Prevenar 13 by 6% to US$ 3.10/dose in poor countries, as part of its commitment to the GAVI global vaccines alliance. This reduction will continue to 2025, safeguarding access against economic growth in developing countries. This announcement came ahead of GAVI’s fundraising conference in Berlin, where it hoped to raise US$ 7.5 billion to extend immunisation in the developing world between 2015 and 2020. GlaxoSmithKline also extended its price–freeze commitment by 10 years for countries moving on from GAVI support, and Sanofi agreed to increase production of yellow fever vaccines to address severe shortages. (*Yahoo news*, 26 Jan 2015)

➢ Harvoni, a drug treatment for Hepatitis C manufactured by Gilead Science, is symbolic of US resentment against expensive drugs, whilst US health care funders take a tougher stance towards cost containment. President Obama’s Affordable Care Act creates incentives to control costs, although direct government intervention is barred. This could cause the US to more aggressively contain spiralling health care costs, of which prescription medicines account for 10% (and rising). The US’s lack of a central mechanism for setting drug prices means that companies charge what the market will bear. However, they argue that this allows them to cover the costs of developing new treatments – with only 4.6% of the world’s population, the US has 33% of global drugs expenditure. Brazil, China, Europe and Japan are all trying to limit costs, and India is issuing patent challenges against western pharmaceutical companies. If other countries reject higher prices, reductions in US prices can only come from industry profit margins – although some commentators believe there is ample scope for this. (*Financial Times*, 10 Feb 2015)

➢ Globally, the Caribbean is one of the regions most affected by HIV, with adult HIV prevalence about 1% higher than any other region except sub–Saharan Africa. Unprotected sex, both heterosexual and between men who have sex with men, are the main routes of transmission, and rates are also high amongst injecting drug users and sex workers. However, stigma and discrimination faced by people infected with HIV drives the epidemic underground, making it difficult to reach many groups. The Antigua and Barbuda HIV/AIDS Network (ABHAN) has a peer/buddy scheme, which recruits, monitors and retains patients into treatment programmes. This had led to decreased risky behaviour and improved health. “Many individuals are reluctant to start treatment because of the myths and stories about HIV and AIDS” says Eleanor Frederick, ABHAN’s director, also stating that equity and social justice are very important in the HIV response. (*IPS*, 10 Feb 2015)

➢ The Puerto Rican Senator, Gilberto Rodriguez, has tabled a bill that would fine parents of obese children up to US$ 800 in an attempt to curb obesity. Children identified as obese will be referred to health advisers to determine the cause of obesity, develop diet and exercise plans with monthly follow–ups. If obesity persists, the parents could be fined up to US$ 500 initially, rising to US$ 800 if there are no improvements. Rebecca Puhl (Rudd Center for Food Policy and Obesity at the University of Connecticut) criticised the proposal for being unfair, an over–simplification of a complex issue and “inappropriately penalises and stigmatises parents.” She states that policies which support parents (eg, improved opportunities for physical activity, more incentives to buy healthier food) are more helpful. (*Yahoo news*, 11 Feb 2015)

➢ The Americas became the first WHO region to eliminate rubella, and the European Region hopes to follow next. Rubella normally produces mild symptoms in children and adults, but it is devastating to foetuses in the first trimester of pregnancy – globally, 120 000 children are born each year with severe birth defects caused by rubella. Rubella is normally prevented by a triple vaccine against measles, mumps and rubella. Endemic measles were eliminated from the hemisphere in 2002, but imported cases have appeared in pockets of unvaccinated children, partly because some parents who believe that the triple vaccine is linked to autism prevent their children receiving it. However, rubella is less contagious than measles, and its vaccine is more effective, so the rare imported cases have not spread so rapidly. “Now with rubella under our belt, we need to roll up our sleeves and finish the job of eliminating measles as well,” says PAHO Director, Dr Carissa Etienne. (*New York Times*, 29 Apr 2015)

